# Whole-body organ-level and kidney micro-dosimetric evaluations of ^64^Cu-loaded HER2/ErbB2-targeted liposomal doxorubicin (^64^Cu-MM-302) in rodents and primates

**DOI:** 10.1186/s13550-015-0096-0

**Published:** 2015-04-14

**Authors:** Daniel F Gaddy, Helen Lee, Jinzi Zheng, David A Jaffray, Thomas J Wickham, Bart S Hendriks

**Affiliations:** Merrimack Pharmaceuticals, One Kendall Square, Suite B7201, Cambridge, MA 02139 USA; STTARR Innovation Centre, Radiation Medicine Program, Princess Margaret Cancer Centre, University Health Network, 190 Elizabeth Street, Toronto, ON M5G 2C4 Canada

**Keywords:** Dosimetry, Copper-64, Positron emission tomography, Nanotherapeutics

## Abstract

**Background:**

Features of the tumor microenvironment influence the efficacy of cancer nanotherapeutics. The ability to directly radiolabel nanotherapeutics offers a valuable translational tool to obtain biodistribution and tumor deposition data, testing the hypothesis that the extent of delivery predicts therapeutic outcome. In support of a first in-human clinical trial with ^64^Cu-labeled HER2-targeted liposomal doxorubicin (^64^Cu-MM-302), a preclinical dosimetric analysis was performed.

**Methods:**

Whole-body biodistribution and pharmacokinetic data were obtained in mice that received ^64^Cu-MM-302 and used to estimate absorbed radiation doses in normal human organs. PET/CT imaging revealed non-uniform distribution of ^64^Cu signal in mouse kidneys. Kidney micro-dosimetry analysis was performed in mice and squirrel monkeys, using a physiologically based pharmacokinetic model to estimate the full dynamics of the ^64^Cu signal in monkeys.

**Results:**

Organ-level dosimetric analysis of mice receiving ^64^Cu-MM-302 indicated that the heart was the organ receiving the highest radiation absorbed dose, due to extended liposomal circulation. However, PET/CT imaging indicated that ^64^Cu-MM-302 administration resulted in heterogeneous exposure in the kidney, with a focus of ^64^Cu activity in the renal pelvis. This result was reproduced in primates. Kidney micro-dosimetry analysis illustrated that the renal pelvis was the maximum exposed tissue in mice and squirrel monkeys, due to the highly concentrated signal within the small renal pelvis surface area.

**Conclusions:**

This study was used to select a starting clinical radiation dose of ^64^Cu-MM-302 for PET/CT in patients with advanced HER2-positive breast cancer. Organ-level dosimetry and kidney micro-dosimetry results predicted that a radiation dose of 400 MBq of ^64^Cu-MM-302 should be acceptable in patients.

**Electronic supplementary material:**

The online version of this article (doi:10.1186/s13550-015-0096-0) contains supplementary material, which is available to authorized users.

## Background

Liposomal encapsulation fundamentally alters the behavior of chemotherapeutics, enabling delivery of potent cytotoxic drugs with an improved therapeutic index. The large particle diameters of liposomes, typically 50 to 120 nm, coupled with varying levels of surface-bound polyethylene glycol (PEG) to limit detection by the mononuclear phagocyte system, result in particles with dramatically different *in vivo* behavior than small molecule therapeutics. It is understood that liposomes accumulate in tissues with functionally porous vasculature, such as the liver and spleen, as well as in tumors with leaky vasculature, via the enhanced permeability and retention (EPR) effect [1-3], while avoiding deposition in tissues with tight endothelial barriers, such as the heart [4]. By contrast, small molecules are passively distributed in and out of almost all tissues in the body, contributing to distinct safety and efficacy profiles.

MM-302 is a HER2-targeted liposomal doxorubicin designed for selective uptake into HER2-overexpressing tumor cells, while minimizing uptake into non-target cells and tissues such as cardiomyocytes. MM-302 was designed to build on PEGylated liposomal doxorubicin (PLD) by retaining the improved cardiac safety profile of PLD relative to conventional doxorubicin and improving anti-tumor activity through immunotargeting of HER2. The mechanism of action of MM-302 comprises deposition of the nanoparticle in tumors with leaky vasculature through the EPR effect, followed by specific targeting and uptake by HER2-overexpressing tumor cells [4-6]. Preclinical studies have demonstrated improved efficacy for MM-302 relative to untargeted PLD [4,6] and quantitatively characterized the relationship between HER2 antigen expression level and MM-302-mediated doxorubicin delivery [5]. Importantly, the conjugation of targeting moieties to liposomes has little effect on gross biodistribution [6,7], provided that the target is not expressed on vascular endothelial cells or ubiquitously expressed in non-target tissues, and liposomal surface properties are not significantly altered. MM-302 is currently in clinical development.

We hypothesize that for liposomal therapies, tumor deposition is highly variable and a rate-limiting step for effective drug delivery and anti-tumor activity [8]. The extent to which liposomes accumulate within tumors is governed by the inherent tumor physiology, as well as the size and surface characteristics of the liposomes. Vascular permeability is highly variable between different tumor types, among patients with the same type of tumor, and between distinct tumors within an individual subject. Using ^111^In-diethylene triamine pentaacetic acid (DTPA)-labeled PEGylated liposomes, Harrington et al. demonstrated that liposome deposition varied from undetectable to 53% ID/kg in patients [9]. Moreover, liposomal tumor deposition has been identified as a rate-limiting step for drug delivery to tumor cells, supporting the theory that deposition variability may directly result in differential therapeutic response [8]. Correspondingly, Arrieta et al. demonstrated that increased deposition of ^99m^Tc-labeled PLD predicted response of mesothelioma patients treated with PLD and cisplatin [10]. This is further supported by preclinical studies showing a direct correlation between variable liposome deposition in rat xenograft models and tumor response to PLD [11]. These results suggest that identification of patients exhibiting increased tumor deposition may improve response to nanotherapeutics.

We recently described the development of a gradient-loadable chelator, diacetyl 4,4′-bis(3-(N,N-diethylamino)propyl)thiosemicarbazone (4-DEAP-ATSC), as a means to efficiently incorporate copper-64 (^64^Cu) into MM-302 [12]. In that work, we demonstrated that ^64^Cu remains stably associated with the liposome following injection into mice or incubation in human plasma. The ability to directly and stably radiolabel nanotherapeutics such as MM-302 offers the possibility of adapting these molecules for combined therapy and diagnostic imaging and provides a valuable translational tool to obtain quantitative biodistribution and deposition data for therapeutic agents. The current study leverages PET/CT imaging in rodents and primates, in addition to traditional rodent organ activity counts, to evaluate the biodistribution and pharmacokinetics of ^64^Cu-MM-302 and estimate human ^64^Cu-MM-302 dosimetry. Results support the selection of a starting radiation dose for a clinical study utilizing ^64^Cu-MM-302 with PET/CT in patients with advanced breast cancer (NCT01304797). This approach offers potential for evaluating the biodistribution of liposomal agents and identifying patients most likely to respond to nanotherapeutics.

## Methods

### Liposome preparation

MM-302 was prepared as previously described [4,6]. MM-302 is a 100-nm liposome formulation composed of hydrogenated soy phosphatidylcholine (HSPC; Lipoid, Newark, NJ, USA), cholesterol, and 1,2-distearoyl-sn-glycero-3-phosphoethanolamine-n-[amino(polyethyleneglycol)-2000] (PEG-DSPE; Avanti Polar Lipids, Alabaster, AL, USA) in a 3:2:0.3 molar ratio. Lipids were hydrated with 250-mM ammonium sulfate, generating an electrochemical gradient for loading doxorubicin (2 mg/mL). Anti-HER2 F5-PEG-DSPE conjugates were produced and inserted into pre-formed, doxorubicin-loaded liposomes, as previously described [13,14]. The extraliposomal buffer was exchanged with 10-mM HEPES-buffered saline (HBS, pH 6.5) via tangential flow filtration (Spectrum Labs; Rancho Dominguez, CA, USA).

### ^64^Cu chelation and liposome loading

The gradient-loadable ^64^Cu chelator, 4-DEAP-ATSC, has been described previously [12]. ^64^CuCl_2_ was obtained from the cyclotron facility at Washington University School of Medicine (St. Louis, MO, USA). ^64^Cu, supplied in 0.1 M HCl, was added to the 4-DEAP-ATSC solution (12 MBq/nmol of 4-DEAP-ATSC). Chelation efficiency was determined after 1 min incubation at room temperature using instant thin layer chromatography (ITLC) developed in 0.1 M citrate buffer (pH 6.0). Under these conditions, unchelated free ^64^Cu travels with the solvent front, while the chelated ^64^Cu:4-DEAP-ATSC complex remains at the sample origin. The ITLC strip was cut into two equal fractions, and the radioactivity of each fraction was measured in a gamma-counter (Perkin Elmer, Waltham, MA, USA). This procedure yields chelation efficiencies consistently greater than 98%.

For loading into liposomes, an aliquot of ^64^Cu:4-DEAP-ATSC (0.167 mol.% of phospholipid) was transferred to liposomes formulated in HEPES-buffered saline solution (pH 6.5). The mixture was heated for 10 min at 65°C, then cooled in an ice water bath. Loading efficiency was determined by size-exclusion chromatography using illustra NICK columns (GE Healthcare Biosciences, Pittsburgh, PA, USA). Importantly, loading of ^64^Cu:4-DEAP-ATSC into liposomes had no effect on the physiochemical properties of the liposomes [12].

### Mouse studies

Mouse studies complied with Institutional Animal Care and Use Committee guidelines. Female CD-1 mice were purchased from Charles River Laboratories (Wilmington, MA, USA). Mice received intravenous injections of 6 MBq of ^64^Cu-MM-302 in a volume of 200 L (*n* = 3 mice per time point). This corresponds to 20 mol/kg phospholipid or 3 mg/kg doxorubicin (drug content basis). An additional mouse was injected and housed separately for serial PET/CT imaging. At the indicated times, mice were euthanized by isoflurane inhalation followed by immediate cardiac puncture. Tissues were collected and weighed, and their activities assessed via gamma-counter. Radiopharmacokinetic parameters were estimated using MATLAB (The Mathworks, Natick, MA, USA) and a non-compartmental model.

### Absorbed radiation dose calculations

The time-integrated activity of each organ was calculated by integrating the area under the time-activity curve using the trapezoidal method [15]. To estimate mean absorbed doses in humans, the relative organ mass scaling method was utilized. The mean absorbed dose in each tissue was derived from the radionuclide concentration, assuming a homogeneous distribution of the radionuclide within any source region. The calculated mean percentage injected dose per gram tissue (%ID/g) for each organ in mice was extrapolated to determine the organ uptake in a 70-kg adult using the following formula:

$$ \left[{\left(\frac{\%}{{\mathrm{g}}_{\mathrm{organ}}}\right)}_{\mathrm{animal}}\times \kern0.5em {\left({\mathrm{kg}}_{\mathrm{Total}\ \mathrm{Body}\ \mathrm{Weight}}\right)}_{\mathrm{animal}}\right] \times {\left(\frac{{\ \mathrm{g}}_{\mathrm{organ}}}{{\mathrm{kg}}_{\mathrm{Total}\ \mathrm{Body}\ \mathrm{Weight}}}\right)}_{\mathrm{human}} = {\left(\frac{\%}{\mathrm{organ}}\right)}_{\mathrm{human}} $$

Extrapolated percent injected activities in human organs were fit with biexponential kinetic models and integrated to estimate the number of disintegrations in source organs. Radiation absorbed doses for each tissue were calculated from the number of disintegrations using OLINDA/EXM software (Vanderbilt University; Nashville, TN, USA) [16].

### Autoradiography

Female CD-1 mice (*n* = 2) were injected intravenously with 6 MBq of ^64^Cu-MM-302. At 21 h post-injection, mice were sacrificed and perfused with 20 mL PBS to remove vascular radioactivity. Both kidneys were collected from each mouse, one dissected along the midline in the coronal direction and the other in the axial direction. Kidneys were fixed in OCT block and immediately processed for cryo-sectioning. Thirteen sections at 100 μm were collected for each kidney for autoradiography. Kidney sections on glass slides (without coverslip) were placed against Super Resolution Storage Phosphor Screens (Perkin Elmer, Waltham, MA, USA), separated by a sheet of plastic wrap, and developed in a tight-sealed cassette for 24 h. Autoradiography images were acquired using Cyclone Plus Phosphor Imager and Optiquant software (Version 5.0; Perkin Elmer, Waltham, MA, USA) at 600 dpi resolution.

### Primate studies

All primate studies were carried out at MPI Research (Mattawan, MI, USA), in compliance with guidelines established by the United States Department of Agriculture Institutional Animal Care and Use Committee. Two female non-naïve squirrel monkeys were obtained from the MPI Research stock colony. Animals were injected intravenously via the saphenous vein with 37 to 55 MBq of ^64^Cu-MM-302 in 5 mL, corresponding to 20 mol/kg (phospholipid basis) or 3 mg/kg (doxorubicin basis).

### Micro-PET/CT imaging

For image acquisition, mice were anesthetized using 2% inhaled isoflurane and air mixture. PET data was acquired on a microPET Focus 220 preclinical scanner (Siemens; Malvern, PA, USA). At the center field of view, the nominal acquisition resolution was 1.4 mm. Static PET image acquisitions were performed consisting of a 45-min emission scan immediately followed by an 8-min transmission scan. The transmission scan, used for attenuation and scatter correction, was acquired using a rotating ^57^Co source. Data were reconstructed using Siemen’s 3D ordered subset expectation maximization and maximum *a posteriori* (OSEM3D/MAP) algorithm with two iterations for OSEM3D and 18 iterations for MAP. The reconstructed image dataset has a voxel size of 0.146 × 0.146 × 0.796 mm. To facilitate co-registration of the PET and CT datasets, mice were kept on the same imaging bed (Equipment Veterinaire Minerve, Esternay, France) under anesthesia and transported between the microPET and the microCT scanners. Anatomical CT scans (16-s acquisition) were obtained on a Locus Ultra microCT preclinical scanner (GE Healthcare; Pittsburgh, PA, USA) operating at 80 kVp and 50 mA. The images were reconstructed with an isotropic voxel size of 0.154 mm. PET and CT images were registered using Inveon Research Workplace (IRW) software (Siemens; Malvern, PA, USA). Mice remained under anesthesia for the duration of the PET and CT image acquisitions (approximately 1 h total imaging time).

Primates were anesthetized with ketamine, and anesthesia was maintained with 2% to 3% isoflurane in 100% oxygen. PET images were obtained at 0 to 1 and 24 h (60-min scan duration), followed immediately by CT scans. PET data were acquired as 6 × 10 min continuous bed scans on a microPET Focus 120 preclinical scanner (Siemens; Malvern, PA, USA) equipped with PET detectors comprised of crystals of 1.5 × 1.5 × 10 mm. Data were reconstructed using a 2D ordered subset expectation maximization (OSEM2D) algorithm. The reconstructed image dataset has a voxel size of 0.866 × 0.866 × 0.796 mm. Animals were maintained on the same bed under anesthesia for transfer between PET and CT instruments. Anatomical CT scans were obtained on a NanoSPECT/CT (Bioscan; Washington, DC, USA) at 80 kVp and 100 A. The images were reconstructed via filtered back-projection with 0.442 × 0.442 × 0.442 mm voxel size. PET and CT images were registered and quantified using VivoQuant software (inviCRO; Boston, MA, USA). Animals remained under anesthesia for the duration of the PET and CT image acquisitions.

### PET/CT image registration and analysis

Mouse PET/CT images were registered using a semi-automated rigid image registration algorithm on an IRW workstation. Regions of interest (ROIs) were drawn manually on PET/CT slices in each organ and tissue of interest. A linear interpolation algorithm was applied to connect the ROIs to generate tissue volumes for quantification. CT data were used to generate contours in regions with low PET signal or poor contrast with respect to adjacent structures. Specifically for the renal pelvis contours, where the spillover effect is present due to high local ^64^Cu concentrations, ROIs were drawn based on the PET signal only and the ^64^Cu-labeled liposome uptake was quantified as %ID rather than %ID/g.

Primate PET data were registered to the corresponding CT data using a rigid (rotation, translation) registration with Mattes mutual information as the metric [17]. ROI analysis was performed for the heart, kidneys, and renal pelvis. Fixed-volume ellipsoid ROIs were positioned within the heart and kidney regions. This approach was chosen for these organs as they are relatively uniform in size and shape and the use of fixed volume ROIs eliminates variability in volume to produce more consistent concentration estimates. Because of its more complex shape and relatively higher variability, renal pelvis ROIs were hand-drawn with a 3D ROI tool (VivoQuant, inviCRO).

### Kidney micro-dosimetry model

Kidney micro-dosimetry was performed using mouse and primate images. The origin of the radioactive focus was determined to be the renal pelvis. The radioactive signal intensities of the entire kidney and renal pelvis were determined from the signal in the left kidney and scaled to reflect the signal in both kidneys, assuming similar microdistribution. Based on autoradiography and PET/CT images, the radioactive focus was exclusively localized within the renal pelvis. The remaining kidney signal was estimated to be uniformly distributed across the other sub-regions (papillae, medulla, and cortex). Time-activity curves were generated from five imaging times (5 min, 2, 8, 21, and 43 h) for mice and two imaging times (0 to 1, 24 h) for primates. Mouse time-activity curves were scaled to human using the organ-weight scaling method and published kidney compartment sizes [18].

The first primate PET/CT image acquisition (0 to 1 h) was binned into six frames of 10 min each to obtain information on early ^64^Cu-MM-302 pharmacodynamics. A kinetic model was constructed to estimate the time-activity curves in the primate kidney. The pharmacokinetics of ^64^Cu were assumed to be consistent with the behavior of MM-302 [4], following mono-exponential kinetics, characterized by central (blood) compartment volume, *V*_d_, and elimination rate constant, *k*el. Blood flow (*Q*) to the kidney transported ^64^Cu into the vascular space of the kidney, the volume of which was characterized by the vascular volume fraction (VVF). Kidney blood flow was assumed to be 5.5 mL/min/kg [19]. Kidney volume was measured to be approximately 5 cm^3^, based on CT analysis. ^64^Cu was assumed to deposit and washout from the tissue space with first-order kinetics, characterized by rate constants *k*in and *k*out, respectively. From the tissue space, ^64^Cu can then accumulate in the renal pelvis, characterized by first-order rate constant, *k*rpu. The renal pelvis volume was characterized by the pelvis volume fraction (PVF). Renal pelvis accumulation was assumed to be irreversible to enable a conservative absorbed radiation estimate of the contribution from the renal pelvis. Data from the blood, total kidney, and renal pelvis sub-region were used to simultaneously fit *V*_d_, *k*el, VVF, *k*in, *k*out, *k*rpu, and PVF parameters, respectively, to the data extracted from each set of primate images using MATLAB Simbiology (The Mathworks, Natick, MA, USA). Model equations and complete parameter descriptions are included in (Additional file 1: Table S1 and S2).

## Results

### Radiopharmacokinetics and biodistribution of ^64^Cu-MM-302 in mice

**Figure 1:**
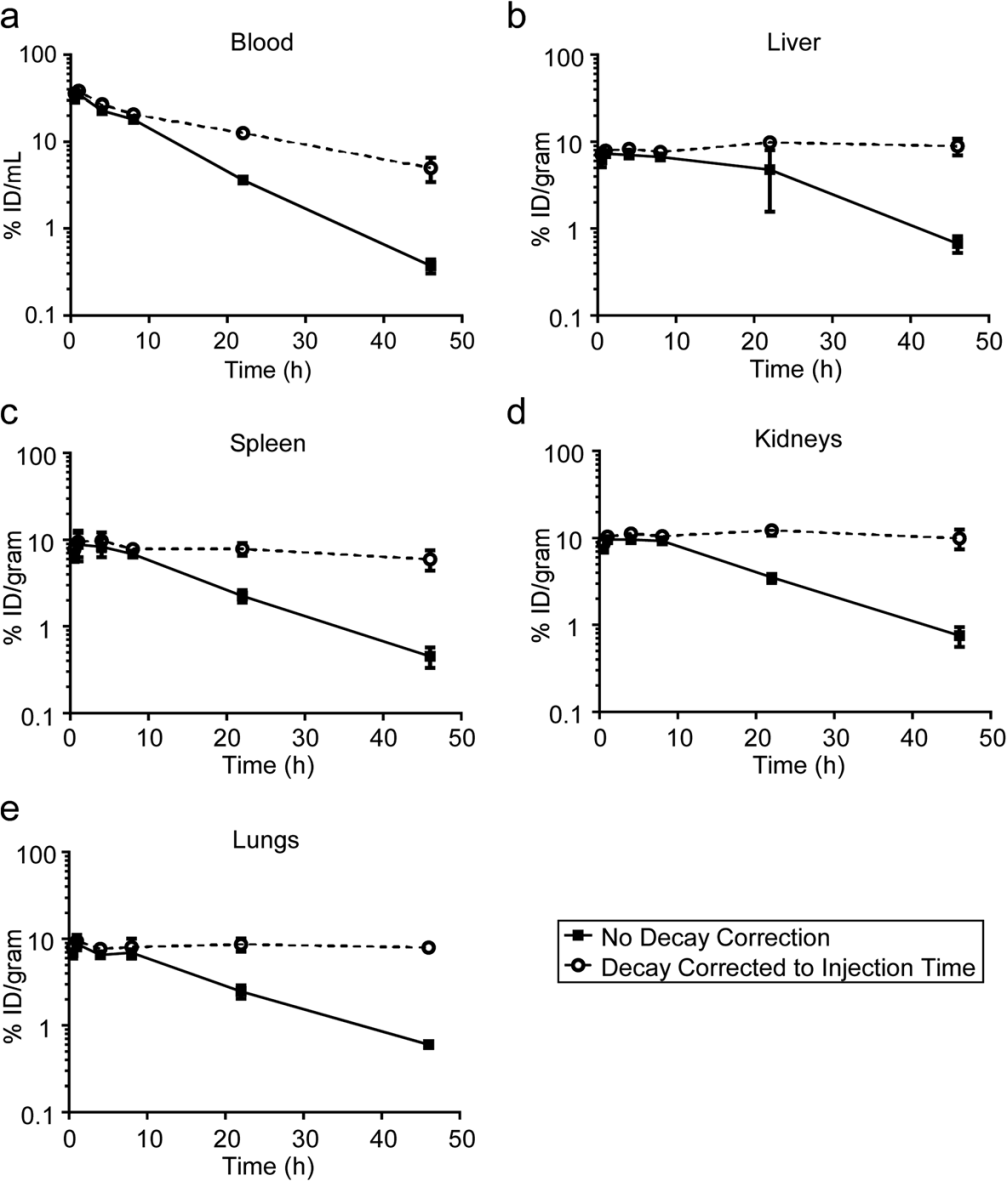
Radiopharmacokinetics of ^64^Cu-MM-302 in blood and maximum exposed organs of CD-1 mice. Data are expressed as %ID/g for the blood **(a)**, liver **(b)**, spleen **(c)**, kidneys **(d)**, and lungs **(e)**. Data represent the mean ± SD of the three mice. Solid lines indicate data that are not decay corrected, representing ^64^Cu decay and ^64^Cu-MM-302 liposome kinetics. Dashed lines represent data that are decay corrected to the time of injection, illustrating the pharmacokinetics of ^64^Cu-MM-302 liposomes.

Radiopharmacokinetic data for ^64^Cu-MM-302 in naïve female CD-1 mice (*n* = 3) are presented in Figure 1a. Radiopharmacokinetic parameters were assessed using non-decay corrected data and are summarized in Table 1. The maximum radioactivity (*C*_max_) in the blood was 35.40 ± 1.86 %ID/g, with an effective ^64^Cu half-life (*t*_1/2_) of 6.81 ± 0.58 h and a clearance rate (CL) of 0.29 ± 0.01 mL/h. Decay corrected data were also utilized to estimate pharmacokinetic parameters of MM-302 liposomes, indicating a vascular *t*_1/2_ of 18.34 ± 2.90 h and CL of 0.13 ± 0.02 mL/h (Additional file 2: Table S3). These results are consistent with published properties of PEGylated liposomes and previous studies measuring the pharmacodynamics of MM-302 [4,20-23].

**Table 1:** **Radiopharmacokinetic parameters of** ^**64**^ **Cu-MM-302**

**Parameter**	**Blood**
*t*_1/2_ (h)	6.81 ± 0.58
*T*_max_ (h)	1.00 ± 0.00
*C*_max_ (%ID/g)	35.40 ± 1.86
CL (mL/h)	0.29 ± 0.01
*V*_ss_ (mL)	2.96 ± 0.18
AUC_(0−∞)_ (%ID/g*h)	347.90 ± 5.63
MRT_(0−∞)_ (h)	10.31 ± 0.77

*Abbreviations:* *t*_1/2_ effective radioactive half-life, *T*_max_ time to reach maximum concentration, *C*_max_ maximum concentration, *CL* clearance rate, *V*_ss_ apparent volume of distribution at steady state, *AUC*_*(0−∞)*_ area under the time-activity curve from 0 h to infinity, *MRT* mean residence time*.*

Complete biodistribution data for ^64^Cu-MM-302 is presented in Additional file 2: Table S4. Most tissues reached *C*_max_ between 1 and 4 h post-injection. The liver, spleen, kidneys, and lungs were the organs with the greatest ^64^Cu-MM-302 accumulation. Time-activity curves were derived from the biodistribution data and are presented for the maximum exposed organs in Figure 1b,c,d,e. Organ time-integrated activity coefficients, equivalent to the number of disintegrations per unit radioactivity (MBq-h/MBq), were calculated using the extrapolated values for human organs by integrating the area under the time-activity curves. Radiation absorbed-dose estimations for human tissues following ^64^Cu-MM-302 administration were determined using OLINDA/EXM and are presented in Table 2. Due to the long circulation time of ^64^Cu-MM-302, the heart was the maximum exposed organ, with an absorbed dose of 0.525 mSv/MBq.

**Table 2:** **Absorbed radiation dose estimates for** ^**64**^ **Cu-MM-302**

**Organ**	**Absorbed dose (mSv/MBq)**
Adrenals	0.049
Brain	0.004
Breasts	0.011
Gallbladder wall	0.017
LLI wall	0.019
Small intestine	0.027
Stomach wall	0.016
ULI wall	0.007
Heart wall	0.525
Kidneys	0.067
Liver	0.059
Lungs	0.053
Muscle	0.009
Ovaries	0.022
Pancreas	0.027
Red marrow	0.014
Osteogenic cells	0.039
Skin	0.004
Spleen	0.049
Testes	0.003
Thymus	0.033
Thyroid	0.005
Urinary bladder wall	0.005
Uterus	0.030
Total body	0.015
EDE	0.061
ED	0.023

EDE, effective dose equivalent; ED, effective dose.

### Mouse PET/CT imaging and kidney microdosimetry

**Figure 2:**
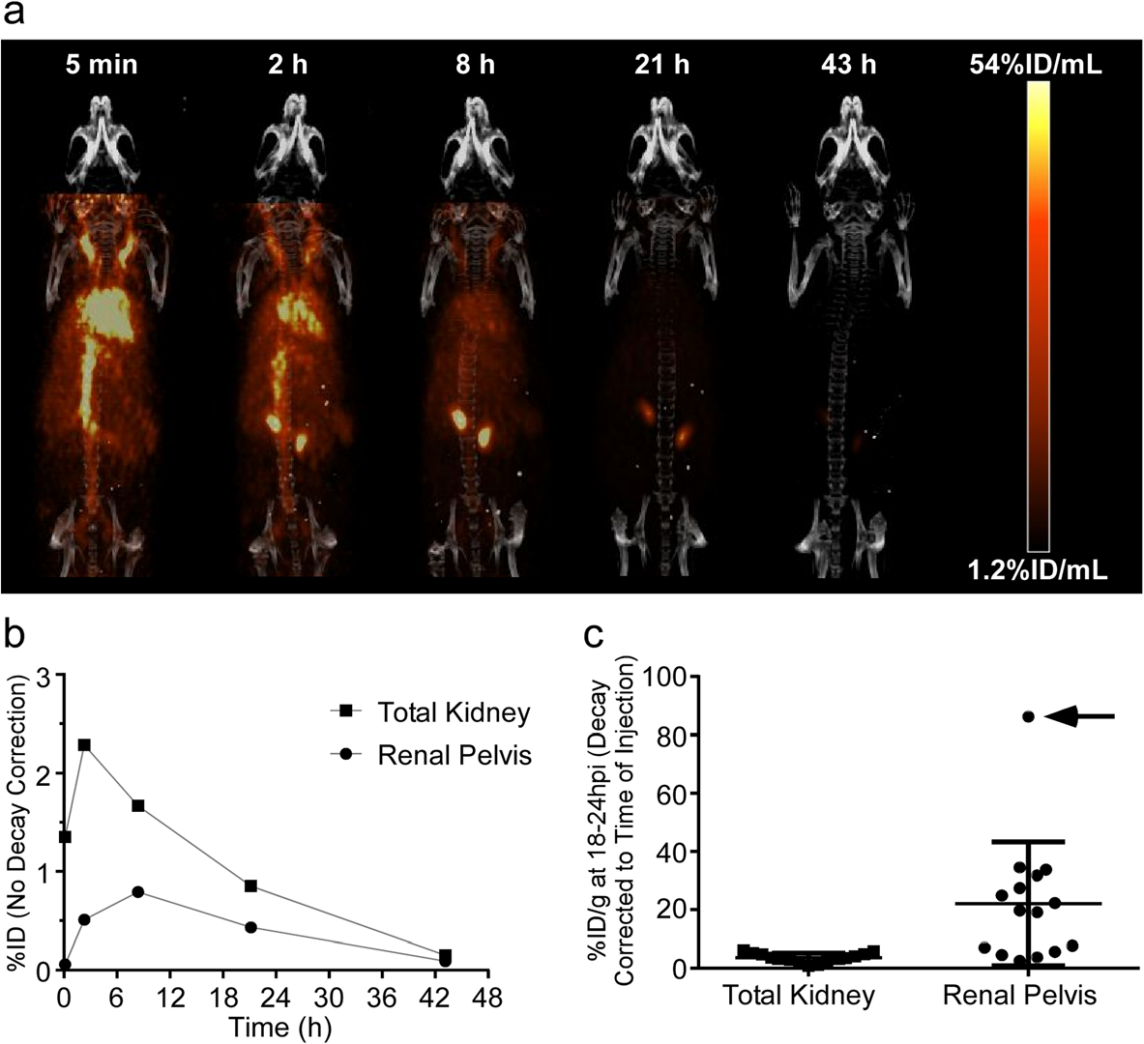
Rodent PET/CT imaging and kidney micro-dosimetry. **(a)** Maximum intensity projection (MIP) PET imaging time-course of a mouse treated with ^64^Cu-MM-302. Data are not decay corrected. **(b)** Time-activity curves of the total kidney (squares) and renal pelvis (circles) generated from the mouse PET images. Data are not decay corrected. **(c)** Comparison of %ID/g in the total kidney and renal pelvis from mice treated with ^64^Cu-MM-302 in independent imaging studies. Each data point represents an individual mouse (*n* = 15). Lines indicate the mean ± SD. The arrow denotes the mouse imaged during the ^64^Cu-MM-302 dosimetry study. Data are decay corrected to the time of injection.

The dosimetry calculations in Table 2 assume a homogeneous distribution of radionuclide within each organ. Inspection of PET/CT images acquired from a mouse injected with ^64^Cu-MM-302 (Figure 2a), however, indicated a non-uniform distribution of the ^64^Cu signal within the mouse kidney. All other organs demonstrated uniform signal distribution. Within the kidney, a focus of ^64^Cu activity appeared in the center of the kidney at early image acquisition times, peaking at 8 h post-injection. Autoradiography studies confirmed the ^64^Cu locus to be the renal pelvis (Additional file 3: Figure S1).

To determine the effect of renal pelvis radioactivity on dosimetry estimations, micro-dosimetry analysis was performed on the kidneys based on the MIRD pamphlet no. 19 kidney micro-dosimetry model [18]. Figure 2b depicts time-activity curves of the total kidney and renal pelvis generated from the PET/CT images at the indicated time points for the mouse depicted in Figure 2a. Human extrapolations were performed as described in the ‘Methods’ section, and radiation absorbed-dose estimates for the kidney sub-regions are presented in Table 3.

**Table 3:** **Absorbed radiation dose estimates for** ^**64**^ **Cu-MM-302 based on mouse and monkey kidney micro-dosimetry**

**Kidney sub-region**	**Mouse (mSv/MBq)**	**Squirrel monkey (mSv/MBq)**
Papillae	0.11	0.22
Pelvis	0.61	0.65
Medulla	0.05	0.16
Cortex	0.04	0.15
Total kidney	0.07	0.17

Figure 2c summarizes the results of multiple independent studies in which mice (*n* = 15) were imaged following injection with ^64^Cu-MM-302. The imaging times were 18 to 24 h post-injection, with a median imaging time of 21 h. It is interesting to note that renal pelvis accumulation was not a ubiquitous phenomenon following ^64^Cu-MM-302 treatment. Background renal pelvis signal, indicating homogeneous distribution throughout the kidney, was observed in 40% (6/15) of mice imaged, while the mouse imaged during the dosimetry study (Figure 2c, arrow) exhibited the highest levels.

### Primate PET/CT imaging and kidney micro-dosimetry

**Figure 3:**
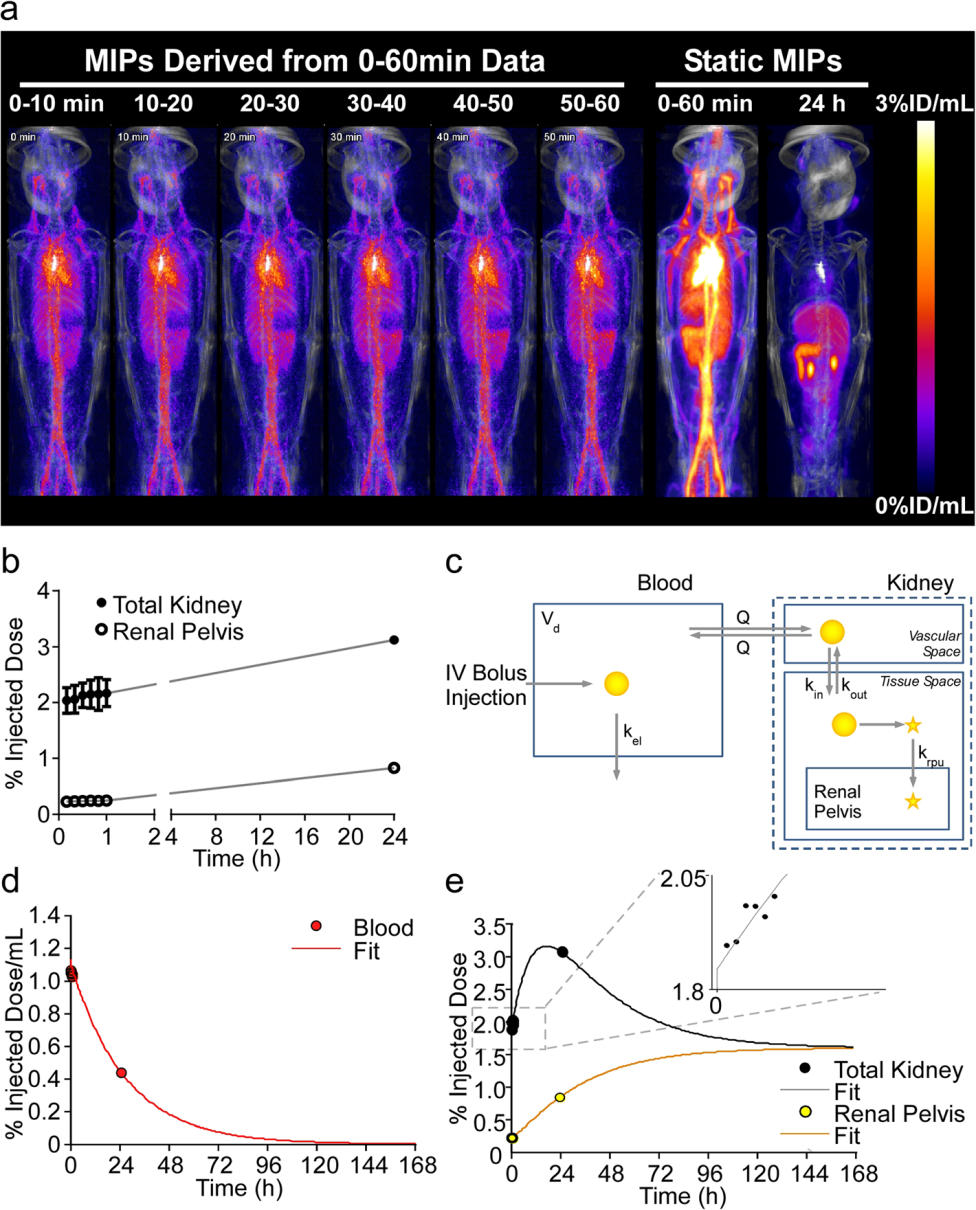
Primate PET/CT imaging and kidney micro-dosimetry. **(a)** MIP PET imaging time-course of a squirrel monkey treated with ^64^Cu-MM-302. Data from 0- to 1-h time-point was segmented into 10-min windows. **(b)** Time-activity curves of the total kidney (filled circles) and renal pelvis (open circles) generated from the primate PET/CT images. **(c)** Schematic representing the physiologically based pharmacokinetic model that was utilized to estimate the full dynamics of ^64^Cu signal from primate images. **(d)** Predicted blood pharmacokinetics of ^64^Cu-MM-302 based on data obtained at the imaging time points (red circles) fit to the primate pharmacokinetic model (red line). **(e)** Predicted kinetics of ^64^Cu accumulation based on data obtained in the total kidney (black circles) and renal pelvis (yellow circles) fit to the primate model for the total kidney (black line) and renal pelvis (yellow line). The inset illustrates accumulation of ^64^Cu signal in the total kidney over the 0- to 1-h imaging time, with each point representing one of the 10-min windows. All data are decay corrected to the time of injection.

The unexpectedly high levels of ^64^Cu accumulation in the mouse renal pelvis following administration of ^64^Cu-MM-302 led to additional kidney micro-dosimetry studies in primates. Primate studies allowed the determination of whether renal pelvis accumulation may be rodent-specific, and the larger primate physiology enabled more accurate kidney micro-dosimetry calculations. Primate PET/CT images were obtained at 0 to 1 and 24 h post-^64^Cu-MM-302 administration (Figure 3a). The first PET/CT image was segmented into 10-min windows of data to provide additional information on early ^64^Cu-MM-302 pharmacokinetics. Intense ^64^Cu activity was observed within the primate kidneys, with the foci assumed to exclusively represent the renal pelvis and the remaining signal uniformly distributed across the other kidney sub-regions. Time-activity curves of the total kidney and renal pelvis were generated from the primate PET/CT images (Figure 3b).

Due to the limited number of primate imaging time points, it was necessary to construct a kinetic model (Figure 3c) to estimate the full dynamics of ^64^Cu uptake in the renal pelvis for accurate estimation of radiation absorbed dose. The model assumed that radioactivity from the blood served as the driving source for accumulation of ^64^Cu in the kidney. ^64^Cu (presumably liposomal, due to absence of bladder signal (Figures 2a and 3a and Additional file 2: Table S4)) deposits and washes out of the kidney tissue space with first-order kinetics, and a portion of the tissue-deposited ^64^Cu may then accumulate in the renal pelvis. Renal pelvis uptake was assumed to be irreversible to enable a conservative absorbed radiation estimate of the contribution from the renal pelvis.

Figure 3d illustrates predicted blood pharmacokinetics of ^64^Cu-MM-302 and indicates a *t*_1/2_ of 20 h for MM-302 liposomes in primates. Figure 3e illustrates the predicted kinetics of ^64^Cu accumulation in the total kidney and renal pelvis. Rather than use organ-weight scaling, the model enabled a physiologically based scale-up to predict human time-activity curves and time-integrated activity coefficients. The following model parameters were changed to reflect human-based values: *V*_d_ was changed to 4.8 L, *k*_el_ was changed to 0.000467 L/min (based on 53 h *t*_1/2_ for PLD) and kidney volume changed to 310 g [24]. Radiation absorbed doses for the kidney sub-regions were calculated using published *S*-values [18] and are presented in Table 3.

## Discussion

We recently reported a method to stably label pre-formed, drug-loaded liposomes with ^64^Cu, providing the opportunity to utilize PET imaging to quantify biodistribution of nanotherapeutics [12]. In the current work, the biodistribution and pharmacokinetics of ^64^Cu-MM-302 were investigated in mice and extrapolated to estimate human dosimetry. Biodistribution was consistent with previously published data, and organ-level dosimetry indicated that the maximum exposed organs were the heart and kidneys, with estimated radiation absorbed doses of 0.525 mSv/MBq and 0.067 mSv/MBq, respectively.

Inspection of mouse PET/CT images revealed a small subregion of high ^64^Cu activity in the renal pelvis. This observation prompted micro-dosimetric evaluation based on imaging data, which measured an absorbed radiation dose of 0.610 mSv/MBq to the renal pelvis. These values were confirmed by analysis of primate imaging data, wherein the estimated absorbed radiation dose to the renal pelvis was 0.650 mSv/MBq.

Multi-scale computational models of liposomal drug delivery to tumors and tissues have previously been developed for PLD and MM-302 [4,8]. Here, we developed and applied a similar kinetic modeling approach to estimate the time-course of ^64^Cu uptake in the total kidney and renal pelvis from the limited set of primate data. Primate micro-dosimetry calculations confirmed mouse data, predicting the renal pelvis to be the maximum exposed kidney sub-region. The model took advantage of known physiological constraints in estimating the time-course of uptake, namely that ^64^Cu should be delivered from the blood. As such, the model took into account the diminishing driving force for kidney deposition as ^64^Cu cleared from the bloodstream over time. Further, the model assumed irreversible deposition of ^64^Cu in the renal pelvis, likely resulting in conservative radiation absorbed-dose estimates. Finally, the model enabled physiologically based scaling to human behavior that may offer more accurate estimations than organ-weight scaling. Expansion of this modeling approach to all organ systems might further improve the ability of preclinical data to accurately predict clinical results. Retrospective analyses would be needed to confirm this hypothesis.

The accumulation of ^64^Cu radioactivity in the renal pelvis was a relatively low fraction of overall administered activity (<2%ID) and, although concentrated in a small area, would not be expected to cause safety concerns. Additionally, the renal pelvis accumulation does not appear to affect levels of deposition in other tissues. While intense kidney signal is not typical of radiolabeled liposomes [25,26], a similar phenomenon has previously been noted in rats treated with ^186^Re-PLD and ^186^Re-PEG-liposomes [27], as well as ^99m^Tc-PLD [28]. Importantly, no accumulation of doxorubicin has been observed in the renal pelvis (unpublished data), further reducing the risk of renal toxicity. With a diameter of approximately 100 nm, the MM-302 liposome is significantly larger than a molecule capable of glomerular filtration [29]. The renal pelvis signal is unlikely to be the result of ^64^Cu pooling in the ureter, as the signal increases relative to background over the 43-h imaging time course, with no significant increase in bladder signal or urine excretion (Figures 2a and 3a, Additional file 2: Table S4 and unpublished data). Mouse studies have demonstrated that >94% of ^64^Cu remains MM-302-associated after 24-h post-injection [12]. This might suggest that ^64^Cu released during clearance of ^64^Cu-MM-302 has affinity for the renal pelvis. However, imaging studies with free ^64^Cu and ^64^Cu:4-DEAP-ATSC, as well as ^64^Cu:4-DEAP-ATSC-labeled nanoliposomal irinotecan (^64^Cu-nal-IRI) have revealed uniform kidney distribution (unpublished data), further suggesting that renal pelvis accumulation is not driven by the chelator.

Biodistribution of ^64^Cu-MM-302 was similar to published data on ^64^Cu-DOTA-labeled PEGylated liposomes [25] and ^111^In-DTPA-labeled PEGylated liposomes in mice [26]. The consistent biodistribution profiles of radiolabeled liposomes suggests that this data may also be used to provide first-order estimates of the radiation absorbed doses for liposomes stably labeled with other radioisotopes. Moreover, while additional factors such as spatial resolution and method of labeling must be considered, such first-order dosimetry estimates may be used to aid identification of optimal isotopes for specific imaging purposes. An example comparison of the liver absorbed-dose estimates for a variety of PET and SPECT isotopes, extrapolated from ^64^Cu-MM-302 mouse dosimetry data, is presented in Additional file 4: Table S5 and suggests that, with regard to organ dosimetry, ^64^Cu is the preferred PET radioisotope for labeling long-circulating liposomes.

Results presented here support the radioactive dose selection of ^64^Cu-MM-302 for clinical studies and illustrate the potential of combining nanotherapeutics with diagnostic imaging. For a proposed starting administered activity of 400 MBq, our analyses predict a radiation dose to the renal pelvis of approximately 250 mSv (25 cGy). These values are in the range of radiation doses reported for several approved radiopharmaceutical agents (Additional file 4: Table S6). ^64^Cu-MM-302 is currently being studied in patients with advanced breast cancer (NCT01304797).

This work represents a step in the development of a liposomal imaging agent for identifying patients with a clinically favorable biodistribution and tumor deposition profile that would be likely to respond to liposomal therapy. A non-therapeutic imaging agent would have the additional advantage of sparing patients harmful side-effects of chemotherapy-containing liposomes during the treatment planning phase. As such, we are currently developing a drug-free ^64^Cu-liposomal PET agent that could potentially be implemented as a companion diagnostic to prospectively select patients for liposomal nanotherapeutics.

## Conclusions

Here, we present murine whole-body biodistribution and pharmacokinetics of ^64^Cu-MM-302, as well as rodent and primate kidney micro-dosimetry, with biodistribution data extrapolated to predict human ^64^Cu-MM-302 dosimetry. Organ-level and kidney micro-dosimetry estimates in rodents and primates support a proposed starting administered activity of 400 MBq of ^64^Cu-MM-302 for clinical imaging studies in patients with advanced HER2-positive breast cancer (NCT01304797). Clinical translation of ^64^Cu-MM-302 PET/CT imaging will determine the extent to which liposomal tumor deposition is predictive of patient response and potentially pave the way for liposome-based imaging diagnostic strategies.

## Additional files

Additional file 1:
**Model equations and complete parameter descriptions.** **Table S1.** Equations for kinetic model of ^64^Cu signal in the kidney arising from liposomal ^64^Cu. **Table S2.** Kinetic model parameter descriptions. Additional file 2: Table S3. Pharmacokinetic parameters of MM-302 liposomes. **Table S4.** Biodistribution of ^64^Cu-MM-302 in CD-1 mice. Additional file 3: Figure S1. Autoradiography was performed to qualitatively measure relative 64Cu intensity in mouse kidney sections. The left image indicates the transversal slice and the right image indicates the coronal slice. Additional file 4:
**Comparison of isotopes useful for radiolabeling liposomes and absorbed radiation dose comparisons for molecular imaging agents.** **Table S5.** Comparison of isotopes useful for radiolabeling liposomes - estimated absorbed radiation dose in liver, based on mice injected with ^64^Cu-MM-302. **Table S6.** Absorbed radiation doses in primary target organs, compared for ^64^Cu-MM-302 and approved molecular imaging agents.
