# Supplementary material for: Whole-body organ-level and kidney micro-dosimetric evaluations of 64Cu-loaded HER2/ErbB2-targeted liposomal doxorubicin (64Cu-MM-302) in rodents and primates
Source: EJNMMI Res. 2015 Apr 14;5:24. doi: 10.1186/s13550-015-0096-0 (PMC4404468; doi:10.1186/s13550-015-0096-0)
Supplement: Additional file 1: — Model equations and complete parameter descriptions. Table S1. Equations for kinetic model of 64Cu signal in the kidney arising from liposomal 64Cu. Table S2. Kinetic model parameter descriptions. [file 13550_2015_96_MOESM1_ESM.pdf]

Table S1: Equations for kinetic model of ^{64}Cu signal in the kidney arising from liposomal ^{64}Cu

State Variable	Equation
Total volume, kidney	$V_{kidney,total} = V_{cap} + V_{tissue}$
^{64}Cu concentration, blood	$\frac{d[Lipo64Cu]_{blood}}{dt} = -k_{el} \cdot [Lipo64Cu]_{blood} - Q \cdot [Lipo64Cu]_{blood} + Q \cdot [Lipo64Cu]_{cap} \cdot \frac{V_{cap}}{V_{blood}}$
^{64}Cu concentration, kidney vascular space	$\frac{d[Lipo64Cu]_{cap}}{dt} = Q \cdot [Lipo64Cu]_{blood} \cdot \frac{V_{blood}}{V_{cap}} - Q \cdot [Lipo64Cu]_{cap} - k_{in} \cdot [Lipo64Cu]_{cap} + k_{out} \cdot [Lipo64Cu]_{tissue} \cdot \frac{V_{tissue}}{V_{cap}}$
^{64}Cu concentration, kidney tissue	$\frac{d[Lipo64Cu]_{tissue}}{dt} = k_{in} \cdot [Lipo64Cu]_{cap} \cdot \frac{V_{cap}}{V_{tissue}} - k_{out} \cdot [Lipo64Cu]_{tissue} - k_{rpu} \cdot [Lipo64Cu]_{tissue}$
^{64}Cu concentration, renal pelvis	$\frac{d[64Cu]_{rp}}{dt} = k_{rpu} \cdot [Lipo64Cu]_{tissue} \cdot \frac{V_{tissue}}{VF_{rp} \cdot V_{kidney,total}}$
^{64}Cu total signal, total kidney	$64Cu_{kidney,total} = [Lipo64Cu]_{cap} \cdot V_{cap} + [Lipo64Cu]_{tissue} \cdot V_{tissue} + [64Cu]_{rp} \cdot VF_{rp} \cdot V_{kidney,total}$
^{64}Cu total signal, renal pelvis	$64Cu_{rp,total} = [64Cu]_{rp} \cdot VF_{rp} \cdot V_{kidney,total} + \left([Lipo64Cu]_{cap} \cdot V_{cap} + [Lipo64Cu]_{tissue} \cdot V_{tissue}\right)VF_{rp}$

The model was implemented in MATLAB SimBiology R2014A (The Mathworks, Natick, MA). For questions, please contact Bart Hendriks (bhendriks@merrimackpharma.com).

Table S2: Kinetic Model Parameter Descriptions

Parameter	Description	Monkey Value	Human Value	Units	Reference
V_{blood}	Blood volume	0.1	4.875	L	Estimated from 1-compartment PK estimates using imaging data; Lee, Lan Na (1998). "Volume of Blood in a Human". The Physics Factbook.
$V_{kidney,total}$	Total kidney volume	0.00487	0.31	L	Measured from imaging data; PMID: 8378254*
V_{cap}	Kidney capillary volume	$0.34*V_{kidney,total}$	$0.34*V_{kidney,total}$	L	NA
V_{tissue}	Kidney tissue volume	$V_{kidney,total}- V_{cap}$	$V_{kidney,total}- V_{cap}$	L	NA
VF_{rp}	Fraction of total kidney volume that comprises the renal pelvis	0.1	0.1	-	Estimated from data
Q	Blood flow rate into/out of kidneys	$5.52*V_{kidney,total}$	$4*V_{kidney,total}$	L/min	PMID: 8378254
k_{el}	Elimination rate constant for liposome clearance from blood	0.000467	0.000218	1/min	Estimated from data; Doxil package insert
k_{in}	Rate constant for liposomal deposition from kidney capillaries into kidney tissue	2.55E-03	2.55E-03	1/min	Estimated from data
k_{out}	Rate constant for liposomal washout from kidney tissue into kidney capillaries	1.00E-03	1.00E-03	1/min	Estimated from data
k_{rp}	Rate constant for renal pelvis uptake of ^{64}Cu from tissue deposited liposomal ^{64}Cu	2.64E-04	2.64E-04	1/min	Estimated from data

*Tissue density was assumed to be 1 kg/L.
Squirrel monkey weight was assumed to be 0.5 kg.
