# Supplementary material for: Whole-body organ-level and kidney micro-dosimetric evaluations of 64Cu-loaded HER2/ErbB2-targeted liposomal doxorubicin (64Cu-MM-302) in rodents and primates
Source: EJNMMI Res. 2015 Apr 14;5:24. doi: 10.1186/s13550-015-0096-0 (PMC4404468; doi:10.1186/s13550-015-0096-0)
Supplement: Additional file 2: Table S3. — Pharmacokinetic parameters of MM-302 liposomes. Table S4. Biodistribution of 64Cu-MM-302 in CD-1 mice. [file 13550_2015_96_MOESM2_ESM.pdf]

Table S3: Pharmacokinetic Parameters of MM-302 Liposomes

Parameter	MM-302
$t_{1/2}$ (h)	18.34 ± 2.90
T_{max} (h)	1.00 ± 0.00
C_{max} (%ID/mL)	38.32 ± 1.98
CL (mL/h)	0.13 ± 0.02
V_{ss} (mL)	3.37 ± 0.10
$AUC_{(0-\infty)}$ (%ID/mL*h)	772.68 ± 126.26
$MRT_{(0-\infty)}$ (h)	26.07 ± 4.60

Abbreviations: $t_{1/2}$, effective radioactive half-life; T_{max}, Time to reach maximum concentration; C_{max}, maximum concentration; CL, clearance rate; V_{ss}, apparent volume of distribution at steady state; $AUC_{(0-\infty)}$, area under the time-activity curve from 0h to infinity; MRT, mean residence time.

Table S4: Biodistribution of ^{64}Cu-MM-302 in CD-1 mice

Tissue	0.5H	1H	4H	8H	22H	46H
Whole Blood	34.78 ± 3.55	36.29 ± 1.90	21.53 ± 1.14	14.32 ± 0.34	3.80 ± 0.40	0.40 ± 0.13
Kidney	8.41 ± 0.86	9.90 ± 1.04	9.09 ± 0.94	7.32 ± 0.55	3.71 ± 0.50	0.81 ± 0.21
Liver	6.95 ± 1.24	7.50 ± 1.07	6.64 ± 0.44	5.30 ± 0.42	2.95 ± 0.29	0.72 ± 0.16
Lungs	7.78 ± 1.10	9.03 ± 1.58	6.17 ± 0.54	5.48 ± 0.78	2.60 ± 0.45	0.64 ± 0.08
Spleen	7.49 ± 1.23	9.01 ± 3.06	7.85 ± 1.92	5.40 ± 0.37	2.37 ± 0.40	0.48 ± 0.13
Adrenals	7.71 ± 0.56	8.15 ± 0.25	6.63 ± 1.36	5.80 ± 3.81	2.06 ± 0.23	0.55 ± 0.08
Heart	5.49 ± 2.04	4.89 ± 0.38	3.74 ± 0.73	2.95 ± 0.43	1.61 ± 0.12	0.37 ± 0.03
Uterus	2.33 ± 0.74	2.79 ± 1.28	3.66 ± 0.62	3.91 ± 2.06	1.60 ± 0.63	0.34 ± 0.11
Ovaries	3.72 ± 0.58	2.93 ± 0.24	4.73 ± 1.15	1.90 ± 0.57	1.43 ± 0.36	0.26 ± 0.04
Gallbladder	15.11 ± 18.39	13.54 ± 13.84	3.75 ± 1.17	3.30 ± 2.06	2.88 ± 1.47	1.17 ± 1.00
Pancreas	2.11 ± 0.08	2.32 ± 0.21	1.39 ± 0.13	1.62 ± 0.14	0.86 ± 0.18	0.19 ± 0.01
Stomach	1.09 ± 0.14	1.17 ± 0.15	1.54 ± 0.14	1.79 ± 0.17	1.40 ± 0.17	0.36 ± 0.01
Small Intestine	1.49 ± 0.34	1.99 ± 0.32	2.61 ± 0.24	3.05 ± 0.55	1.88 ± 0.21	0.43 ± 0.09
Large Intestine	0.83 ± 0.22	1.07 ± 0.21	1.44 ± 0.20	2.31 ± 0.16	1.63 ± 0.36	0.44 ± 0.04
Bladder + Content	1.51 ± 0.39	1.31 ± 0.19	1.06 ± 0.18	0.91 ± 0.45	0.48 ± 0.04	0.15 ± 0.02
Thymus	1.29 ± 0.31	1.92 ± 0.57	1.12 ± 0.23	0.90 ± 0.30	0.54 ± 0.07	0.14 ± 0.01
Bone Marrow	2.18 ± 1.47	2.32 ± 0.54	1.61 ± 0.40	1.67 ± 0.70	0.77 ± 0.13	0.18 ± 0.04
Bone	0.78 ± 0.30	0.74 ± 0.26	0.93 ± 0.10	0.73 ± 0.10	0.21 ± 0.11	0.06 ± 0.02
Muscle	0.57 ± 0.18	0.74 ± 0.03	0.68 ± 0.32	0.64 ± 0.13	0.25 ± 0.01	0.07 ± 0.01
Brain	1.01 ± 0.11	0.87 ± 0.10	0.50 ± 0.03	0.35 ± 0.11	0.14 ± 0.00	0.04 ± 0.01
Skin	0.43 ± 0.10	0.69 ± 0.39	0.99 ± 0.46	0.90 ± 0.07	0.67 ± 0.15	0.18 ± 0.02
White Adipose Tissue	0.63 ± 0.11	0.66 ± 0.22	0.70 ± 0.26	0.72 ± 0.21	0.24 ± 0.10	0.15 ± 0.10
Brown Adipose Tissue	2.19 ± 0.58	3.27 ± 0.75	2.16 ± 0.52	1.24 ± 0.40	1.10 ± 0.05	0.41 ± 0.15

Data are expressed at % injected dose per gram (% ID/g ± SD, n = 3 mice at each time point) and are not decay corrected.
