# Supplementary material for: Whole-body organ-level and kidney micro-dosimetric evaluations of 64Cu-loaded HER2/ErbB2-targeted liposomal doxorubicin (64Cu-MM-302) in rodents and primates
Source: EJNMMI Res. 2015 Apr 14;5:24. doi: 10.1186/s13550-015-0096-0 (PMC4404468; doi:10.1186/s13550-015-0096-0)

Figure S1: Autoradiography was performed to qualitatively measure relative ^{64}Cu intensity in mouse kidney sections. The left image indicates the transversal slice and the right image indicates the coronal slice.

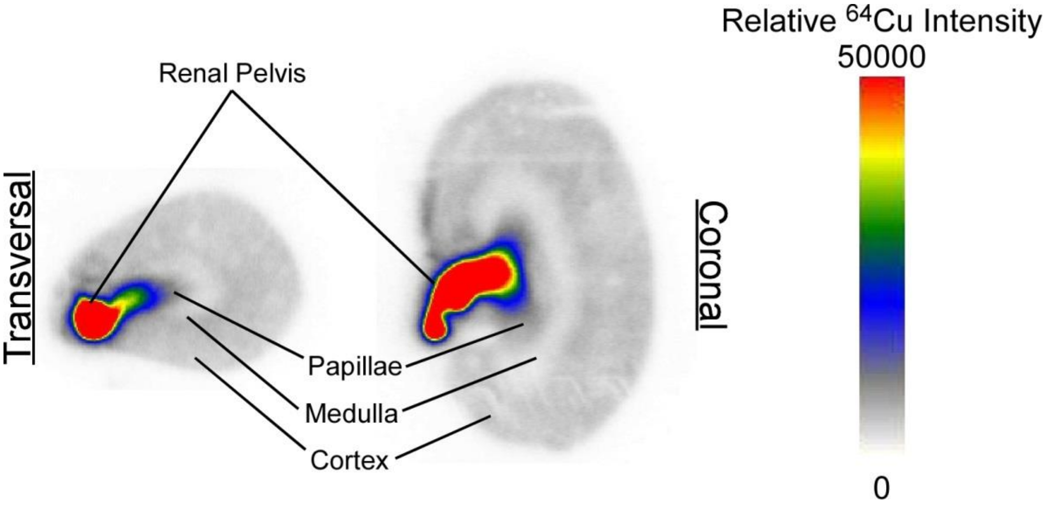

Supplement: Additional file 3: Figure S1. — Autoradiography was performed to qualitatively measure relative 64Cu intensity in mouse kidney sections. The left image indicates the transversal slice and the right image indicates the coronal slice. [file 13550_2015_96_MOESM3_ESM.pdf]
