# Supplementary material for: Whole-body organ-level and kidney micro-dosimetric evaluations of 64Cu-loaded HER2/ErbB2-targeted liposomal doxorubicin (64Cu-MM-302) in rodents and primates
Source: EJNMMI Res. 2015 Apr 14;5:24. doi: 10.1186/s13550-015-0096-0 (PMC4404468; doi:10.1186/s13550-015-0096-0)
Supplement: Additional file 4: — Comparison of isotopes useful for radiolabeling liposomes and absorbed radiation dose comparisons for molecular imaging agents. Table S5. Comparison of isotopes useful for radiolabeling liposomes - estimated absorbed radiation dose in liver, based on mice injected with 64Cu-MM-302. Table S6. Absorbed radiation doses in primary target organs, compared for 64Cu-MM-302 and approved molecular imaging agents. [file 13550_2015_96_MOESM4_ESM.pdf]

Table S5: Comparison of Isotopes Useful for Radiolabeling Liposomes – Estimated Absorbed Radiation Dose in Liver, Based on Mice Injected with ^{64}Cu-MM-302

Isotope	$t_{1/2}$ (h)	Imaging Modality	β- Energy (Abundance)[a]	β+ Energy (Abundance)[a]	γ Energy (Abundance)[a]	Estimated Absorbed Dose (mSv/MBq)[b]	Predicted Necessary Starting Activity (MBq)[c]	Estimated Radiation Dose for Predicted Starting Activity (mSv)[c]
^{18}F	1.8	PET	n/a	634 KeV (97%)	511 KeV (194%)	0.013	1.78e5	2348
^{99m}Tc	6.0	SPECT	293 KeV (100%)	n/a	141 KeV (89%)	0.003	349	1
^{64}Cu	12.7	PET	578 KeV (37%)	653 KeV (18%)	511 KeV (35%)	0.059	400	24
^{111}In	67.3	SPECT	n/a	n/a	245 KeV (94%)	0.241	26	6
^{67}Ga	78.2	SPECT	n/a	n/a	93 KeV (39%)	0.159	134	21
^{89}Zr	78.4	PET	n/a	396 KeV (23%)	909 KeV (99%)	0.811	134	108
^{186}Re	89.2	SPECT	1069 KeV (71%)	n/a	137 KeV (9%)	0.972	260	253
^{124}I	100.3	PET	n/a	1532 KeV (11%)<br>2135 KeV (11%)	603 KeV (63%)	1.320	177	50

[a]Isotope energies were taken from Holland, et al. 2010. Unconventional Nuclides for Radiopharmaceuticals. *Molecular Imaging*, 9(1) and Be, et al. 2013. Table of Radionuclides Vol 7, *Monographie BIPM-5*, and reflect the most abundant transition.
[b]Human absorbed radiation dose estimates for the selected isotopes were extrapolated from ^{64}Cu-MM-302 mouse biodistribution data.
[c]Starting activity for each isotope was predicted based on estimated activity remaining at 24h post-injection with ^{64}Cu-MM-302, adjusted for isotope energy efficiency, and estimate a starting activity that would be required to obtain sufficient image quality after 24h. For example, with a positron abundance of 18%, ^{64}Cu requires approximately 100 MBq of activity at 24h, or 400MBq starting activity, whereas ^{18}F, with a positron abundance of 97%, requires approximately 20MBq of activity at 24h, but a starting activity of approximately 178GBq due to its much shorter physical half-life.

Table S6: Absorbed Radiation Doses in Primary Target Organs, Compared for ^{64}Cu-MM-302 and Approved Molecular Imaging Agents

Status	Agent	Target Organs	Radiation Absorbed Dose (mSv/MBq)	Administered Activity (MBq)	Radiation Dose per Administered Activity (mSv)	Reference
Clinical Trial	^{64}Cu-MM-302	Renal Pelvis	0.61	400	244	Table 3
		Heart Wall	0.53	400	210	Table 2
Approved	BEXXAR (^{131}I-tositumomab)	Thyroid	2.71	185	501	[1]
		Kidneys	1.96	185	363	
		Upper Large Intestines	1.34	185	248	
		Lower Large Intestines	1.30	185	241	
		Heart Wall	1.25	185	231	
Approved	ProstaScint (^{111}In-Capromab pendetide)	Liver	1.00	185	185	[2]
		Spleen	0.88	185	163	
Approved	^{111}In-Oxiquinoline	Spleen	10.81	18.5	200	[3]
Approved	OctreoScan (^{111}In-Pentetreotide)	Spleen	0.67	222	148	[4]
		Kidneys	0.49	222	108	

Information for approved imaging agents was derived from human dosimetry data presented in package inserts:

1. BEXXAR (131I-tositumomab) [Package Insert]. Wilmington (DE): GlaxoSmithKline; 2003.
2. ProstaScint (111In-Capromab Pendetide) [Package Insert]. Langhorne (PA): EUSA Pharma, Jazz Pharmaceuticals; 1996.
3. In-111 Oxyquinoline Solution [Package Insert]. Arlington Heights (IL): Amersham Health, Medi-Physics, Inc.; 1994.
4. OctreoScan (111In-Pentetreotide) [Package Insert]. St. Louis (MO): Mallinckrodt, Inc.; 1995.
